# PTOP and TRF1 help enhance the radio resistance in breast cancer cell

**DOI:** 10.1186/1475-2867-14-7

**Published:** 2014-01-25

**Authors:** Zheng Li, Xiaoxi Yang, Nengxing Xia, Lei Yang, Haijun Yu, Fuxiang Zhou, Conghua X, Yunfeng Zhou

**Affiliations:** 1 Department of Radiation and Medical Oncology, Zhongnan Hospital, Wuhan University, Donghu road 168, 430071, Wuhan City, Hubei Province, China

**Keywords:** PTOP, TRF1, Telomere bonding protein, Telomere, Radio resistant

## Abstract

**Purpose:**

The telomere binding proteins play an important role in telomere function, which contribute greatly to the radio resistant in human cancers. This research is designed to investigate the relationship among the telomere length, telomerase activity and changes of telomere binding protein PTOP and TRF1 in radio resistant breast cancer cell lines.

**Methods:**

Irradiate MDA-MB-435 s breast cancer cell with total dose of 60 Gy delivered in 2 Gy/fraction and 6 Gy/fraction respectively, then measuring their telomere length by Southern blot analysis,telomerase activity by Telomerase PCR Elisa and detecting the expression of PTOP and TRF1 in both gene and protein levels. To further investigate the function of PTOP, using lentivirus technic to silence the PTOP gene and the detected the new silenced cells by southern blot and telomerase activity.

**Results:**

2 radio resistant breast cancer cell lines were successfully established. The MDA-MB-435 s R60/6 was (approximate 8.1-8.6 kbp) about 2–2.4 folds to the patent cell (3.6-4.2 kbp), the MDA-MB-435 s R60/2 cell (approximate 5.3-6.3 kbp) was about 1.3-1.75 fold to the parent cell line. The telomerase activity was more enhanced in radio resistant cell lines than the parent cell. The expression of PTOP and TRF1 were significant increased in radio resistant cell lines than the patent cell in both gene and protein level. Otherwise, after using lentivirus technic to silence the PTOP gene, we found the radio resistant cell lines were significant decrease their radio resistances and telomerase activities.

**Conclusion:**

The telomere binding protein PTOP and TRF1 were increased expressed in radio resistant breast cancer cell, PTOP was observed instinct positive correlated with telomere lengthen and telomerase activity enhancement.

## Background

Telomere, caps the end of the mammalian chromosomes, is composed of repeated “-TTAGGG-’” DNA sequences and telomere binding proteins, serves in the protecting of the degradation of chromosome and intracellular signaling for regulating cell proliferation [1-3]. The telomere binding proteins, also called shelterin complex, contains PTOP, TRF1, TRF2, TIN1, TIN2 and POT1, are essentials for telomere function. Some research claim that the PTOP and TRF1 play an important role in interaction with other proteins and the length regulation also has some relations to the telomerase activity [4,5]. Our previous research demonstrated there were some relationships with telomere length and cellular radio resistance [6]. Furthermore an increase expression of PTOP was observed in a radio resistant human larynx squamous carcinoma [7]. However, this point has not been confirmed in the breast cancer cell lines. This study is designed to investigate the relationships among the telomere length, telomerase activity and the expression of some telomeric proteins in breast radio resistance cells.

## Results

### Establish radio resistant breast cancer cell mode

**Figure 1:**
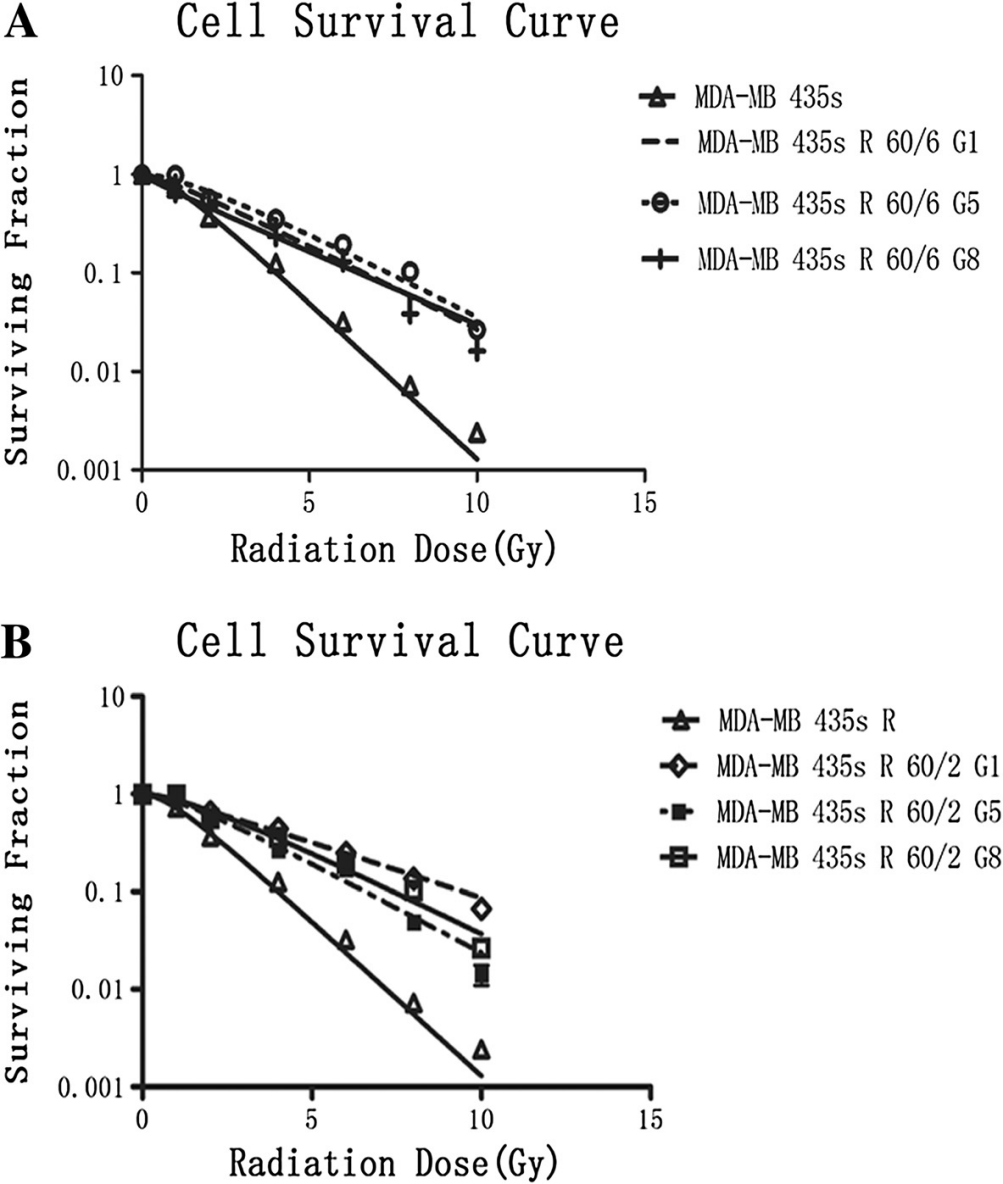
**Clonogenic assay. A** and **B** described the cell survival condition after irradiation. X-ray irradiation was performed as planed in 6 Gy/fraction and 2 Gy/fraction with total dose 60 Gy. An appropriate number of cells in different generations were seed in 6-well plates. Each group was irradiated at the dose of 0 Gy, 1 Gy, 2 Gy, 4 Gy, 6 Gy, 8 Gy, 10 Gy, respectively. After incubation for 14 days, the colonies were fixed and stained by crystal violet. The coloniesâ€‰>â€‰50 cells were scored as viable colonies. The data was analyzed by single-hit multi-target model and survival curve of each group was described by Graphpad prism 5.0 software.

**Figure 2:**
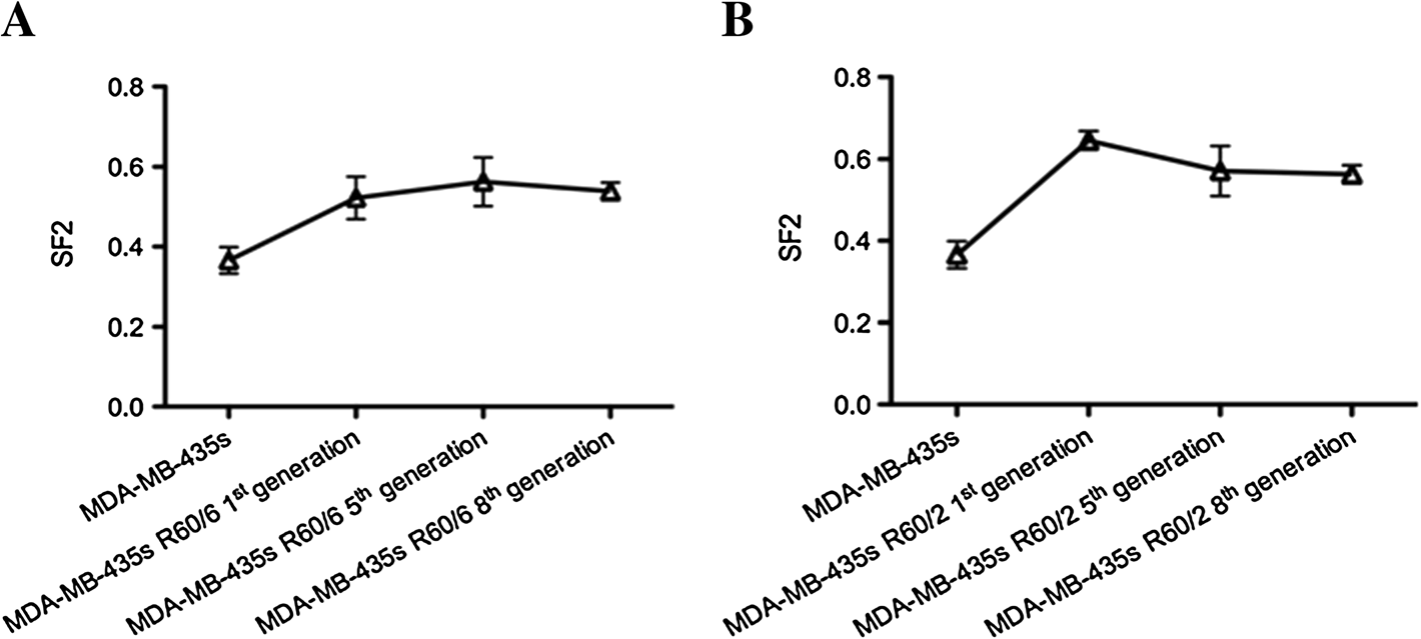
**SF2 in different generation. A** and **B** demonstrated the changes among non-irradiated group and irradiated groups. All the irradiated groups in 2A and 2B were increased greatly than the non-irradiated cell (MDA-MB-435 s). Although a slight decrease were observed along the generation, all the values of SF2 were significant higher than the MDA-MB-435 s cell. The SF2 of parent cell were 0.3655 ± 0.0334, the 1st, 5^th^and 8th generation of 60/6 cell lines were 0.5224 ± 0.0534, 0.5628 ± 0.0611, 0.5387 ± 0.0225, respectively. The 1st, 5th and 8th generation of 60/2 were 0.5224 ± 0.0534, 0.5627 ± 0.0611, 0.4687 ± 0.0225, respectively. Compare with the parent cell line, the 60/6 and 60/2 cell lines all increased their radio resistivity and stay persistent, p < 0.05.

Using X-ray to irradiate the MDA-MB-435 s cell to 60 Gy total dose by 2 Gy/fraction and 6 Gy/fraction respectively. MDA-MB-435 s R60/2 and MDA-MB-435 s R60/6 cells were passaged until the 8th generation. The 1st, 5th and 8th cells were collected for the clonogenic assay. The cell survival curves of Figure 1A and B demonstrated the radiobiological parameters of each groups. The survival fraction of each generation of MDA-MB-435 s R60/6 and MDA-MB-435 s R60/2 were much higher at each point than the MDA-MB-435 s cell. And Figure 2A and B described the changes of SF2 in different generation of irradiated cells. Each generation of irradiated cells were higher than the parent cell and stay relatively stable. The radiobiological parameters calculated in Table 1 showed that, D0, Dq and SF2 values in MDA-MB-435 s, MDA-MB-435 s R60/6 and MDA-MB-435 s R60/2 on average. The Irradiated groups were significant higher than the parent cell line (p < 0.05). The MDA-MB-435 s R60/6 cell and MDA-MB-435 s R60/2 cell were observed as stable and radio resistant cell lines.

**Table 1:** Radiobiological parameters in different groups

**Groupings**	**D0**	**Dq**	**SF2**
MDA-MB-435 s	1.372 ± 0.012	0.413 ± 0.033	0.365 ± 0.008
MDA-MB-435 s R60/2	3.782 ± 0.041*	0.667 ± 0.015*	0.646 ± 0.024*
MDA-MB-435 s R60/6	2.563 ± 0.028*	0.593 ± 0.189*	0.534 ± 0.006*

Data were fit into the single-hit multi-target model, and survival curves of both groups were drawn by Graphpad Prism, the SF2, D0, Dq values were calculated (pD0, Dq, SF2 < 0.05). Data were expressed as the mean ± SD. * p <0.05 were considered significant. D0: mean lethal dose, Dq: quasi-threshold dose, SF: surviving fraction.

### The changes of telomere ponding proteins (PTOP and TRF1)

**Figure 3:**
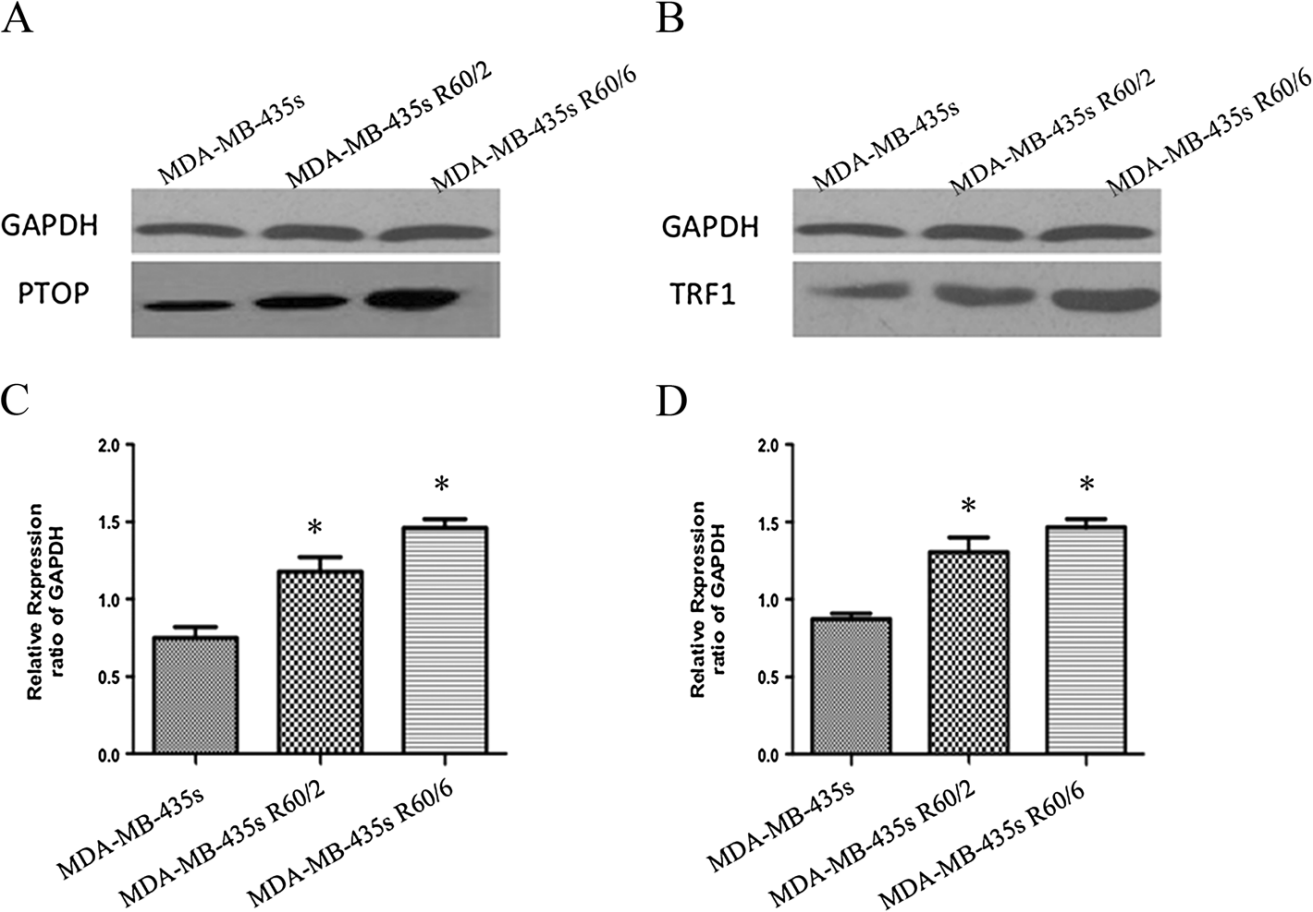
**Changes of PTOP and TRF1 proteins in different groups. (A and B)** Western blot showed the protein of PTOP and TRF1 in irradiated groups were significant over expressed than the non-irradiated group (MDA-MB-435 s). (3C and 3D) The bar chart demonstrated the semiquantitative analysis of PTOP and TRF1 expression. Data represented means ± SD,*p < 0.05 was considered significant.

**Figure 4:**
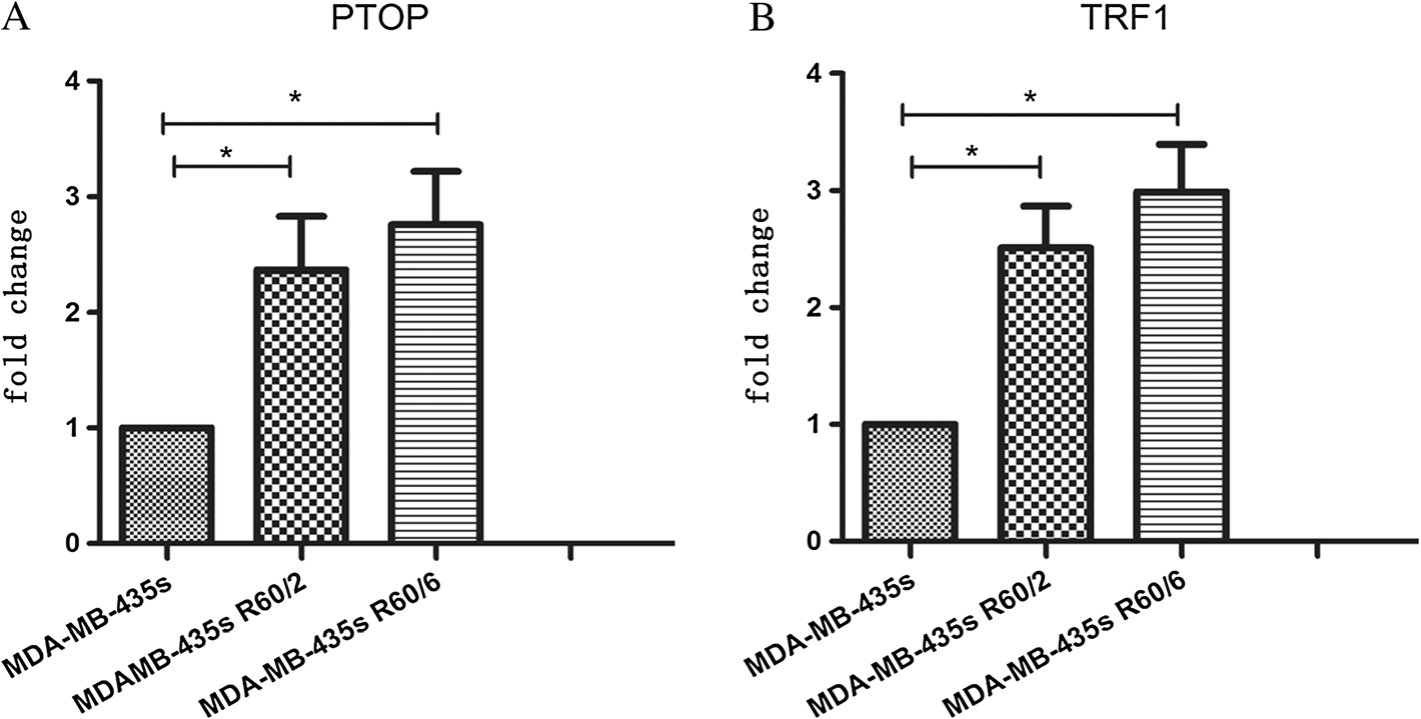
**Changes of PTOP and TRF1 mRNA in different groups.** qTR-PCR was used to evaluated PTOP and TRF1 mRNA expression in non-irradiated and irradiated groups. The results (4A and 4B) showed the relative levels of PTOP and TRF1 mRNA compare with GAPDH. Bar graph showed the mean ± SD value of relative mRNA expression, *p < 0.05 was considered significant.

With an irradiation of 60 Gy, we detected there were some changes in telomere ponding proteins, such as PTOP and TRF1 (Figures 3 and 4). Western blot and qRT-PCR were used to evaluated the changes of PTOP and TRF1 in both protein and mRNA level. Compared with the MDA-MB-435 s group, the expression of irradiated groups were increased in both protein and mRNA level. The results suggested highly expressed PTOP and TRF1 had some relationship with radio resistance.

### The telomere length exam

**Figure 5:**
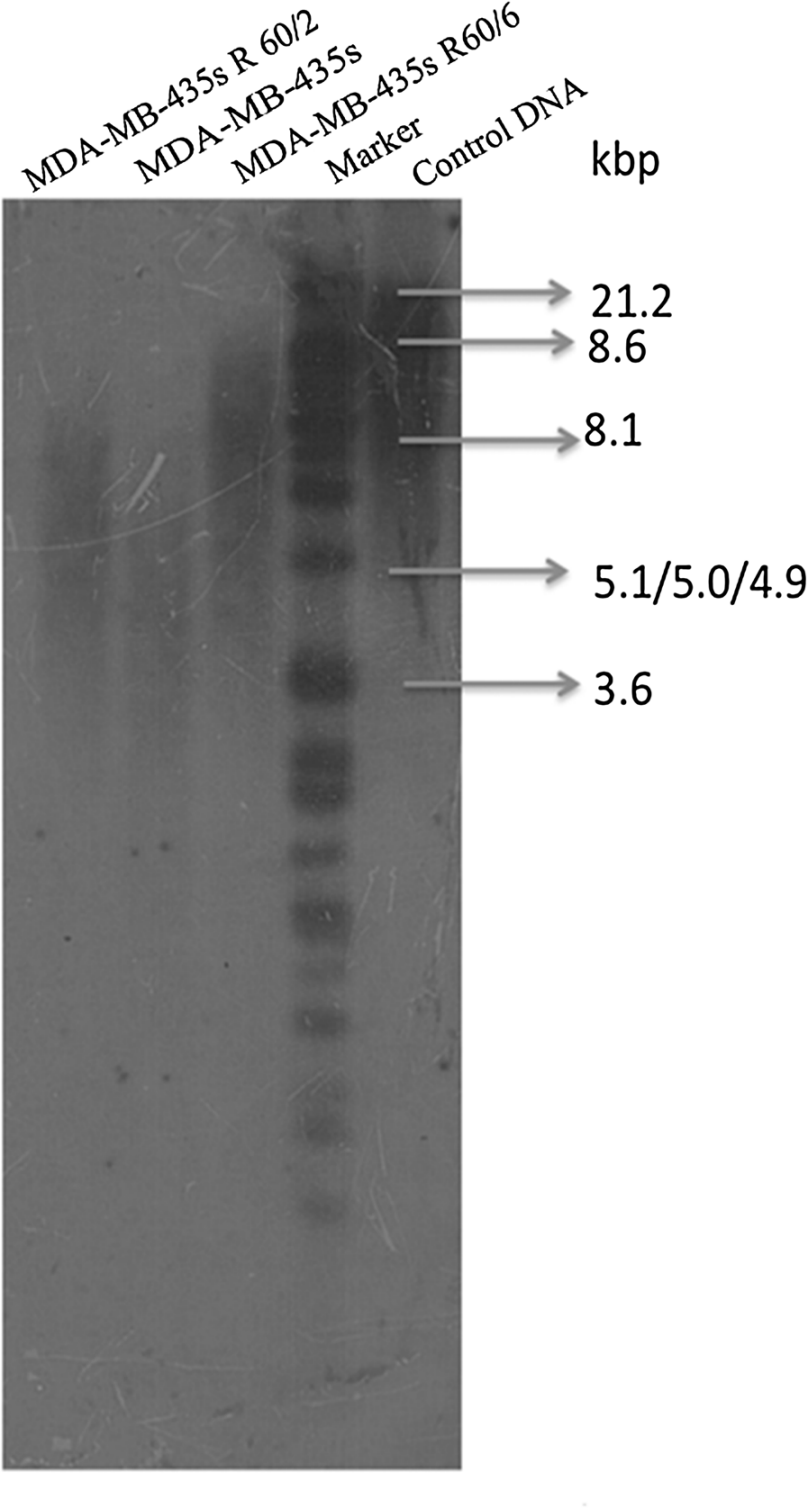
**Southern blot analysis.** Telomere length was detected by Southern blot analysis. The results were analyzed by image system software (Bio-Rad).

To further investigate the relationship between telomere length and telomere ponding protein, Southern blot analysis was performed. In Figure 5, the results showed the MDA-MB-435 s R60/6 and MDA-MB-435 s R60/2 cell lines were significant longer than the parent cell. We calculated the average telomere length of irradiated groups, the mean TRF (terminal restriction fragment) of MDA-MB-435 s R 60/6 cell line (approximate 7.2-8.6 kbp) was about 2–2.4fold to the MDA-MB-435 s cell (Approximate 3.6-4.2 kbp), the MDA-MB-435 s R 60/2 cell line (approximate 5.3-6.3 kbp) was about 1.3-1.75 fold to the parent cell line. These results suggested the PTOP and TRF1 highly expressed groups had longer telomere length than the normal expressed one.

### The results of telomerase activity in different groups

**Figure 6:**
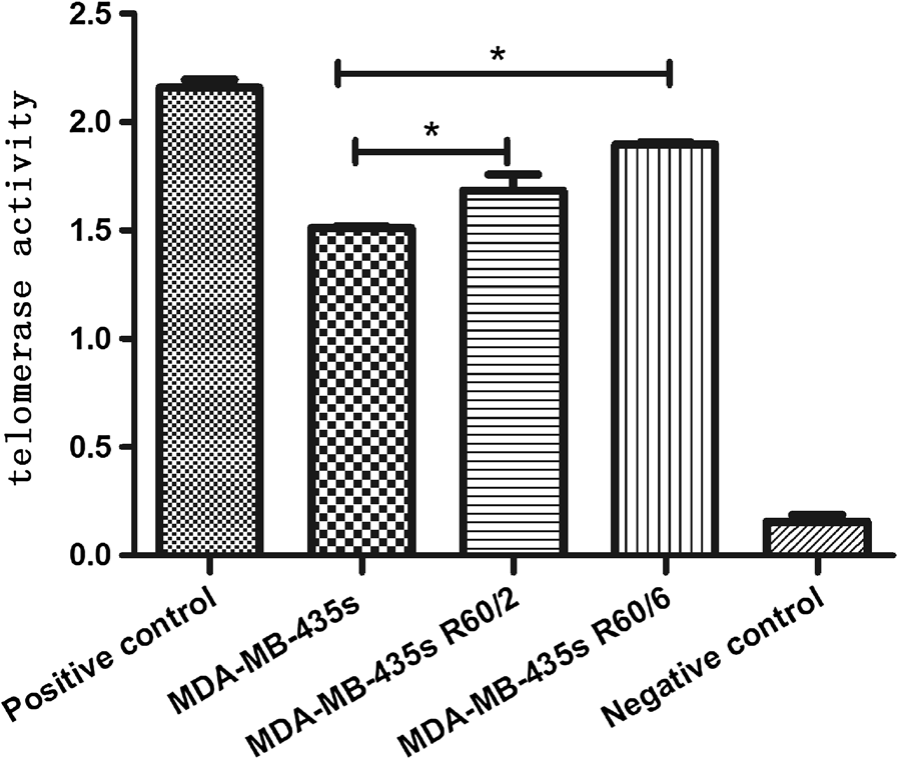
**Telomerase activity in different groups.** The telomerase activity was performed by telomerase activity assay. Compare with the MDA-MB-435 s cell line, the MDA-MB-435 s R60/6 and MDA-MB-435 s R60/6 cell lines had increased telomerase activity.

To investigate whether the elongated telomeres had increased telomerase activity, the cells of 3 groups were detected by telomerase activity assay. Compare with the MDA-MB-435 s cell line, the MDA-MB-435 s R60/6 and MDA-MB-435 s R60/2 cell lines were increased in telomerase activity in Figure 6. *p < 0.05, was considered significant. The highly expressed PTOP and TRF1 groups were observed having increased telomerase activity.

### Verify the effect of PTOP knocking down cell lines

**Figure 7:**
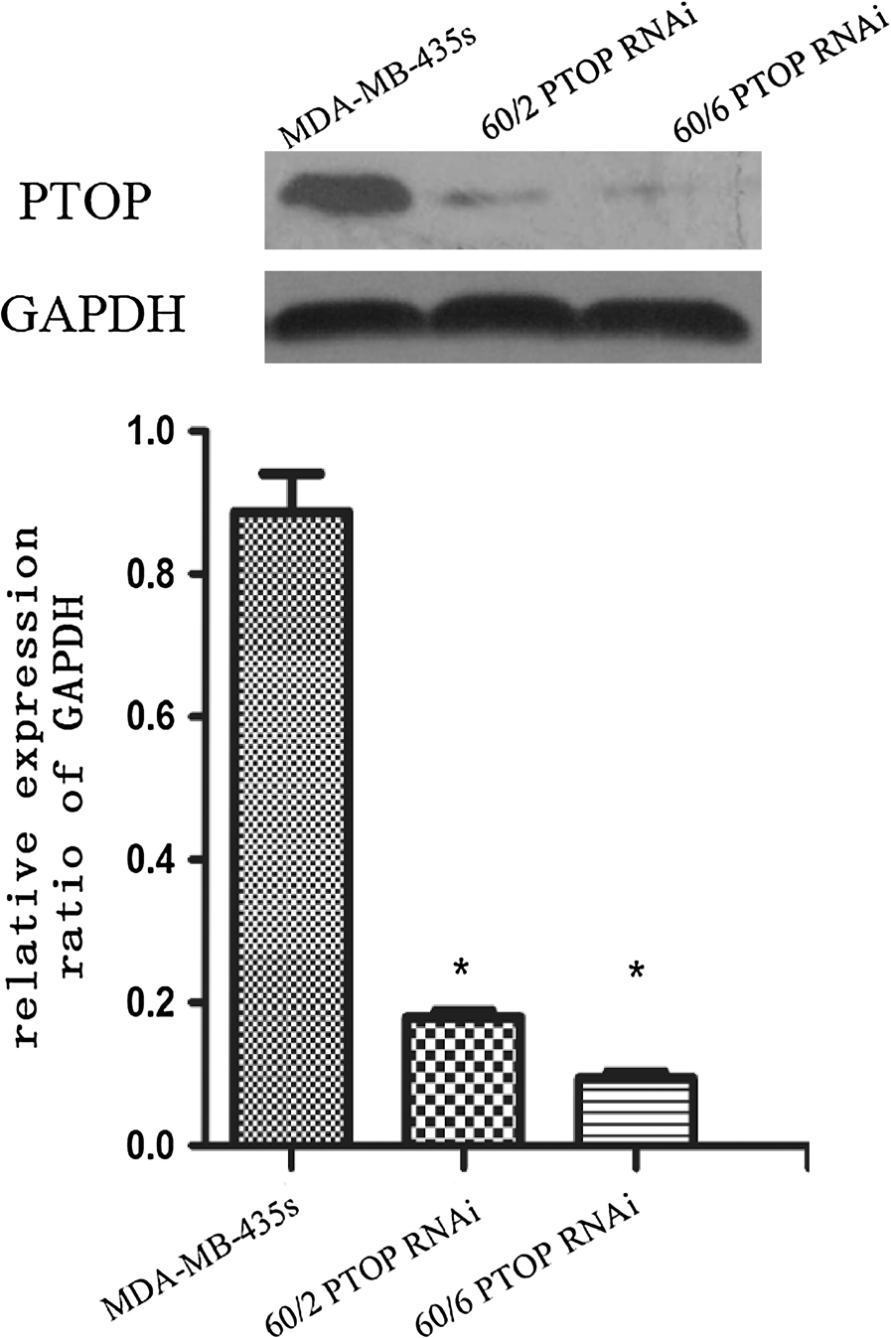
**Knocking down PTOP protein in different groups.** Western blot showed the PTOP knocking down expression in different groups. Compare with the MDA-MB-435 s cell, the expression of 60/2 PTOP RNAi and 60/6 PTOP RNAi cells were significant decreased. The bar graph showed the semiquantitative analysis of PTOP knocking down expression. Data represented means ± SD, *p < 0.05 was considered significant.

**Figure 8:**
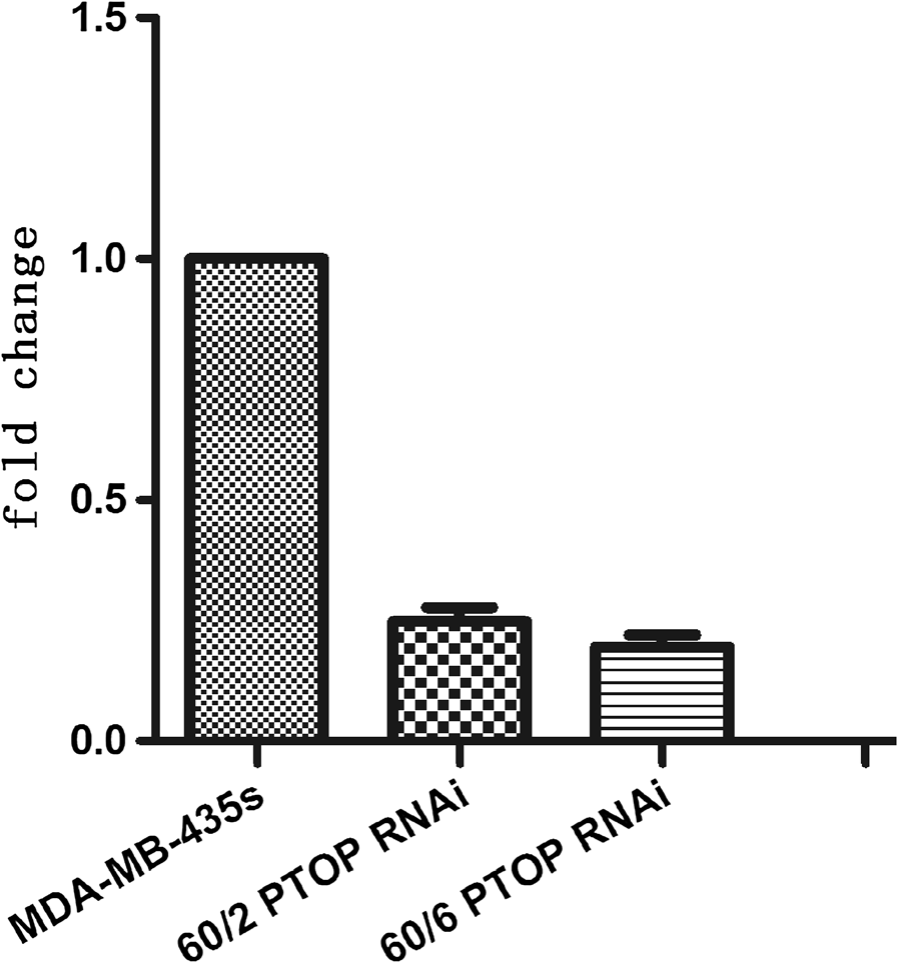
**mRNA expression of Knocking down PTOP in different groups.** The results showed the relative levels of silenced PTOP mRNA compare with GAPDH in 3 cell groups. Bar graph showed the mean ± SD value of relative mRNA expression, *p < 0.05 was considered significant.

Whether PTOP regulate the radio resistance of the breast cancer cell, we knocked downed the PTOP gene by lentivirus technic. To verify the effect of knocking down, western blot and qPT-PCR were performed in MDA-MB-435 s, MDA-MB-435 s 60/6 LV-PTOP-RNAi (60/6 PTOP RNAi for short) and MDA-MB-435 s R60/2 LV-PTOP-RNAi (60/2 PTOP RNAi for short) cell lines. In Figures 7 and 8, compare with the MDA-MB-435 s cell, the expression of 60/2 PTOP RNAi and 60/6 PTOP RNAi cells were significant decreased. These results suggested the new PTOP silenced cell lines were well conducted for further experiments.

### Changes of radio resistance by knocking down PTOP

**Figure 9:**
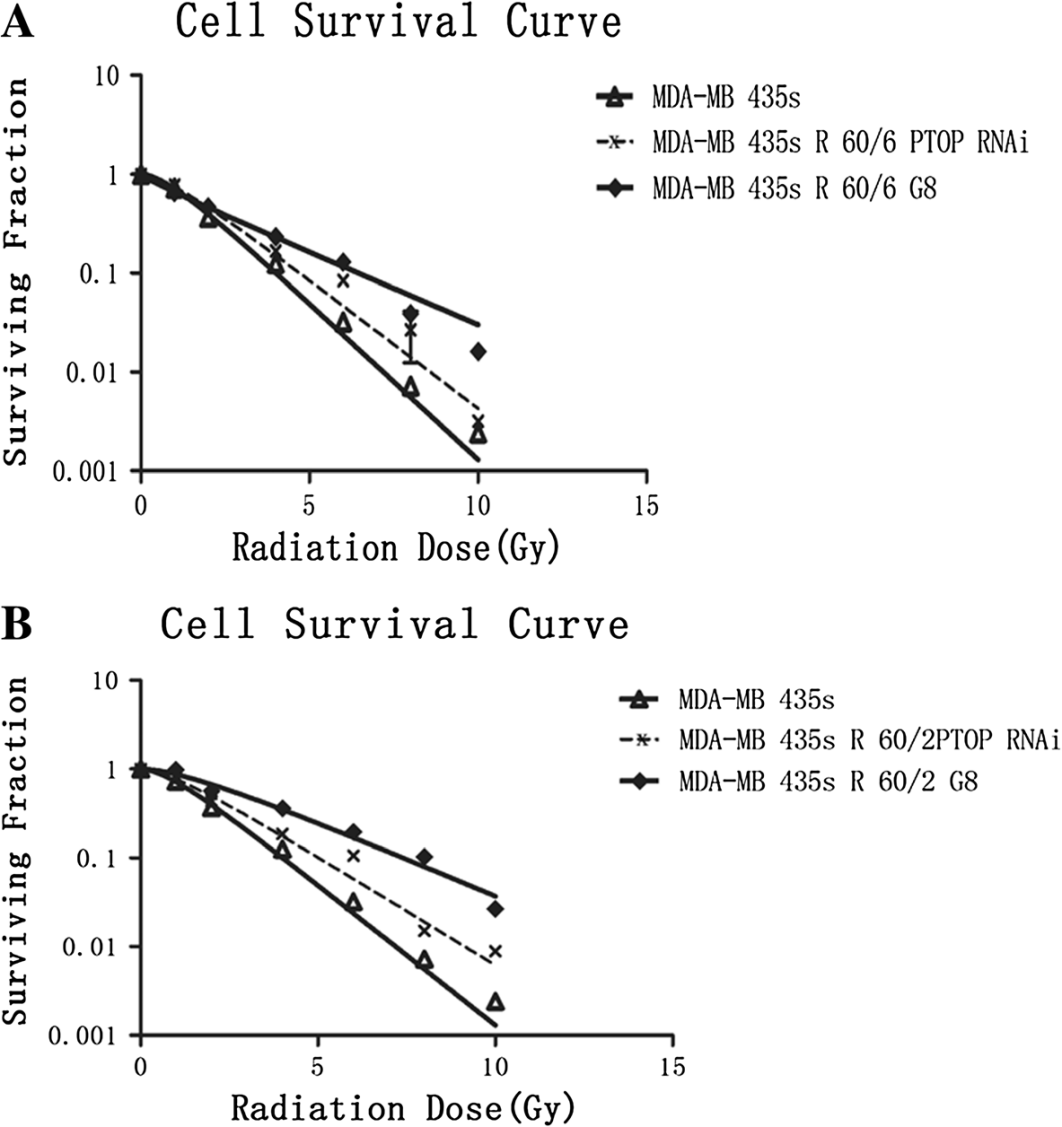
**Changes of radio resistance by knocking down PTOP. A** and **B** showed the different cell survival curves among the MDA-MB- 435s cell, radio resistant cell lines and PTOP silenced cell lines. Each group was irradiated at the dose of 0 Gy, 1 Gy, 2Gy, 4 Gy, 6 Gy, 8 Gy, 10 Gy, respectively. The same procedures were performed as above. The data was analyzed by single-hit multi-target model and survival curve of each group was described by Graphpad prism 5.0 software.

Whether PTOP was the key factor to the radio resistance, the clonogenic assay would clearly tell us on basis of such well-established PTOP silence cell lines. The MDA-MB-435 s, 60/2 PTOP RNAi and 60/6 PTOP RNAi cells were collected and seed in 6-well plates. Followed the same procedures as above, the results suggested 2 PTOP silenced cell lines both decrease their radio resistance but still radio sensitive than the MDA-MB-435 s cell in Figure 9. These results revealed that the existing of PTOP would changes the radio resistance of breast cancer cell.

### Changes of telomere activity of PTOP Knocking down cell lines

**Figure 10:**
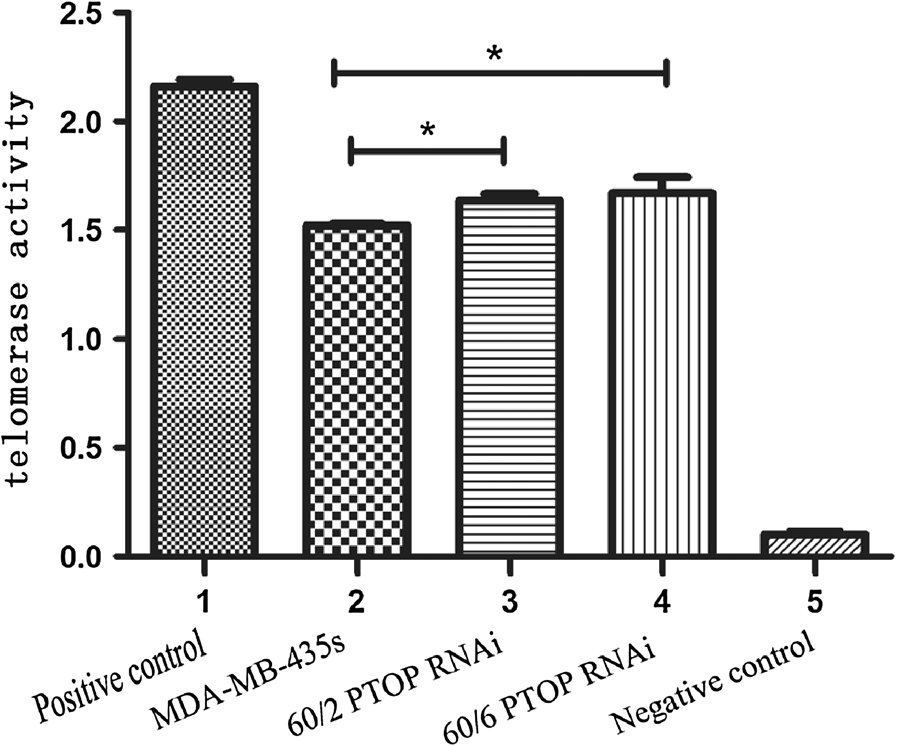
**Changes of telomerase activity by knocking down PTOP.** The telomerase activity was performed by telomerase activity assay. Compare with the MDA-MB-435 s cell line, the PTOP silenced groups had no statistical differences. *pâ€‰>â€‰0.05

After we performed the telomerase activity assay, we found the PTOP also played an important role in telomerase activity. In Figure 10, the bar graph showed compare with the MDA-MB-435 s cell, the PTOP silenced groups were slight increase their telomerase activity, but had no significant differences. Which meant, lacking of PTOP would decrease the telomerase activity.

## Discussion

In the guidelines of the breast cancer, radiotherapy takes a great part in the treatment strategy and influence the prognosis of the patients. How to enlarge the effect of the radiotherapy or avoid the radio resistance? These questions lead us to do this research.

In our study, the telomere binding protein PTOP and TRF1, as the important mediators of telomere maintenance, were significant highly expressed in breast radio resistant cell lines at both protein and gene level. In addition, in the 2 breast radio resistant cell lines, the MDA-MB-435 s R60/6 showed longer telomere length and higher telomerase activity.

These 2 radio resistant cell lines were established by the same parent cell and share the similar tumor characteristic. Their resistances were measured by cell survival curve. Although they maintained resistance, a slight decrease of radio resistance still can be observed in the 5th and 8th generation. Some research explains, because of the absence of the selecting radiation pressure, the radio resistant modes will lose their ability gradually. It is postulated that the radiation pressure has the favored the emergence of radio resistant cell lines during fractionated irradiation [8]. Li [9] et al. irradiate human breast cancer cell MCF-7 in total 40 Gy delivered in 2 Gy per fraction, lost the radio resistance after 12 generations.

In our study, the telomere length and radio resistance were positive correlated. At meanwhile, among these 3 cell lines, we found that the telomerase activity was positive correlated with telomere length. Wesbuer [10] et al. investigate the human neuroblastomas, find out that increase the telomere length would lead to the decreasing of radio sensitivity, and the telomerase negative cells are significant more sensitive to irradiation. The same phenomena are observed in murine lymphoma cell and human primary lung cancer [11,12]. Taken together, these data suggest that telomere length and telomerase activity may be used as a potential diagnostic marker for detecting radio resistance.

TRF1, is engaged in multiple roles at telomere including telomere protection, telomere replication, sister telomere resolution and telomere length maintenance [13]. TRF1 was expected as a negative regulator of telomere length factor, could reduce the activity of telomere at the beginning [14]. However, in our study, not only the gene but also the protein level, it seemed that TRF1 had a positive relationship with the telomere length and telomerase activity. Interestingly,the similar phenomena are observed in acute leukemia cell and pancreatic cancer cells [15,16]. Some research explains because the expression of TRF1 may be related to hTERT (human telomerase reverse transcriptase) expression. Furthermore, the c-MYC gene plays an important role in the promoter region of hTERT, which is widely over expressed in breast cancer. hTERT is up-regulated by c-MYC independently from cell proliferation and negative influenced by TRF1 [17,18]. Therefore, the relationship between TRF1 and telomerase activity might due to other factors, such as hTERT, c-MYC and so on.

PTOP, known as TPP1, is considered directly interact with the telomerase for recruitment to telomere [19], helps in stabilizing the TRF1-TIN1-TRF2 interaction. From the results of our study, we observed that in the radio resistant cell lines, the PTOP was highly expressed. Meanwhile, when we knock downed the PTOP gene of the radio resistant cell lines, a dramatically decrease of radio resistance was appeared and a slight decrease of telomere activities were also observed. Some other research also pointed out, knocking down the PTOP reduced the ability of endogenous TRF1 associate with TRF2 [20]. Although the mechanism of recruitment remains unclear, the results of out study provide the evidence that PTOP had a close relationship with intrinsic radio resistance and telomere activity. Many other research have also demonstrated that the PTOP helps in extending the telomere length and enhance the telomerase activity alone or working with other telomere binding proteins, and even influence the radio sensitivity of other cancer cells. From our study, it was clear that regulating the radio resistance was a huge and complicate process, although PTOP played an extremely crucial role in regulation, other proteins also took parts in the this reaction [21-25].

## Conclusion

Our research provided that the hypo fraction irradiation could establish a more radio resistant breast cancer cell, with longer telomere and more active telomere. High levels of PTOP and TRF1 genes were expressed in the radio resistance breast cancer cells, knocking down the PTOP reduced the radio resistance and the telomere activity. Why we couldn’t block completely the radio sensitivity completely by knockdown the PTOP, are there any other genes interact with this gene. The further research will be designed through this promising direction.

## Methods

### Cell culture

MDA-MB-435 s is a mammary gland breast cancer cell, originally described as a spindle shaped variant of the parental MDA-MB-435 cell. The cells were maintained in RPMI 1640 medium supplemented with 10% fetal bovine serum and incubated at 37°C in 5% CO2/95% air. The cells were sub cultured every 2 to 3 days in vented 25-cm^2^ cell culture flasks.

### X-ray irradiation

MDA-MB-435 s radio resistant cells modes were built in 2 ways. One was MDA-MB-435 s R60/6, the other was MDA-MB-435 s R60/2. All the cells were first grown to approximately 90% confluence then irradiated by a Simens Primus Accelerator at an average dose rate of 2.0 Gy/min, 60/6 were received 6 Gy/fraction; 60/2 were received 2 Gy/fraction. The total dose were 60 Gy respectively.

### Clonogenic assay

The standard clonogenic assay was performed as described [26,27]. Cells were trypsinzed at room temperature for 30-60 sec, pipetted the clumped cells. The single cell suspension was adjusted and seeded into typical 6 flasks. Then, cells were left to settle over night, and exposed to irradiation at room temperature over the dose range 0-10 Gy, followed by incubation at 37°C, 5% CO2 for 14 days. After fixation and staining, colonies of >50 cells were scored. Surviving rates were evaluated relative to 0 Gy radiation treated controls.

### Western blot

All the proteins were isolated from cells by RIPA lysis buffer, separated on SDS-PAGE, and transferred to PVDF membranes. After blocked, the membranes were incubated with first antibodies anti-PTOP and anti-TRF1 (1:1000) at 4°C overnight, then incubated with secondary antibodies (1:1500) at 37°C for 2 h and detected by ECL solution, then tape the membrane to inside film cassette then exposed the membrane to film with x-ray in the dark. Anti-PTOP antibody (ab57595) was from abcam®, Anti-TRF1 antibody (Cat.#04-638) was from Millipore®.

### qRT-PCR

Total RNA was isolated by TRIzol reagent. The analysis was performed as described [28]. The forward and reverse primers of each gene were as follow: GAPDH were 5′-tggaaggactcatgacaca-3′ and 5′-ttcagctcagggatgacctt-3′, PTOP were 5′-atctcgAagtatgcctggccgctgtcagagtg-3′ and 5′-agcggccgctatcacatcggagttggctcagac- 3′, TRF1 were 5′-gcttgccagttgagaacgata-3′ and agggctgattccaagggtg-3′, respectively. All the primers were composed by Invitrogen® company. Each cycle consisted on denaturation at 94°C for 30s, annealing at 58°C for 30s, extension at 72°C for 30s. All the samples were tested by at least 3 times. All the experiments were carried out on Mx3000P (stratagene) and the results was analyzed using the MXP 3000 analysis program.

### Southern blot analysis

Mean telomere length was measured by TRF analysis using the Telo TAGGG Telomere Length Assay Kit(Roche). Each sample was digested with 4 μg DNA by mixture of Hinf I and Rsa I enzymes for at least 2 hours. Then the DNA fragments were electrophoresed by agarose gel and transferred on a nylon membrane by southern blotting. The Blotted DNA fragments were hybridized to a DIG-labeled probe. The mean terminal restriction fragments length was repeats 4 times and valued by image system software (Bio-Rad).

### Telomerase activity assay

The PCR-based telomerase assay was performed as described [29]. In brief, 4 × 10^5^cells were harvested and followed the protocol of Telo TAGGG Telomerase PCR ELISA (Roche).

### Lentivirus and transfection

The knockdown products of LV-PTOP-RNAi was composed by Genechem® company. The target PTOP sequence was 5′- GTGGTACCAGCATCAGCCT-3′, the NC sequence was 5′- TTCTCCGAACGTGTCACG T-3′, respectively. Both MDA-MB 435 s R60/2 and 60/6 cells were seeded in 6-well culture plates at the density of 1 × 10^5^cells/well 24 hours before transfection. In the procedure of transfection, the culture medium was replaced by 2 ml serum-free, antibiotics-free DMEM, the virus solution and polybrene mixture. The cells were incubated for 8-10 h at 37°C in 5% CO2/95% air. Then the medium was completely replaced and continued to incubate for at least 72 h. Then the cells were collected and determinate the expression level of PTOP mRNA by qRT-PCR , the level of PTOP protein by Western Blot.

### Statistical analysis

All the data were obtained from triplicate samples and recorded as the mean ± standard deviation (SD). Statistical analyses were performed by χ^2^ test. Statistical analysis was used by software SPSS 10.0 and Graphpad Prism 5.0 c. P < 0.05 was considered to be statistically significant.

## Abbreviations

TRF1: Telomeric repeat binding factor 1; SDS-PAGE: Sodium dodecyl sulfate polyacrylamide gel electrophoresis; PVDF: Polyvinylidene Fluoride; ECL: Electro-Chemi-Luminescence; NC: Negative control; DMEM: Deulbecco’s Modified Eagle Medium; SD: Standard deviation; hTERT: Human telomerasereverse transcriptase; SF: Survival fraction.

## Competing interests

The authors declare that they have no competing interests.

## Authors' contributions

Dr. ZL, first author, wrote the paper and performed all the experiments. Dr. XX Y, helped in performing telomerase activity assay. Dr. LY, helped in performing southern blot assay. Dr. XN X, helped in performing telomerase activity assay. Dr. HJ Y, helped in revising the paper. Prof FX Z, helped in designing the experiment, control the process and the quality of all the experiments. Prof. CH X, helped in designing this study. Prof. YF Z, corresponding author, the head of National Natural Science Foundation of China, the Doctoral Fund of Ministry of Education of China and the Fundamental Research Funds for the Central Universities, helped in designing the experiment, control the process and quality of the experiments. All authors read and approved the final manuscript.

## Authors’ information

Dr. Zheng LI: 3rd grade of Ph.D. in department of radiation and medical oncology of Zhongnan Hospital of Wuhan University.2010.11-2011.11, working as an intern in the Department of Radiotherapy of Grenoble Central Hospital of Joseph-Fourier University in France. Dr. Xiaoxi Yang, Ph.D. in Department of Radiation and Medical Oncology of Zhongnan Hospital of Wuhan University. Dr. Lei YANG, Ph.D. in Department of Radiation and Medical Oncology of Zhongnan Hospital of Wuhan University. Dr. Nengxing XIA, Ph.D. in Department of Radiation and Medical Oncology of Zhongnan Hospital of Wuhan University. Dr. Haijun YU, doctor in Department of Radiation and Medical Oncology of Zhongnan Hospital of Wuhan University. Prof. Fuxiang ZHOU, vice-chief of Department of Radiation and Medical Oncology of Zhongnan Hospital of Wuhan University. Prof. Conghua XIE, chief of Department of Radiation and Medical Oncology of Zhongnan Hospital of Wuhan University. Prof. Yunfeng ZHOU, president of Zhongnan Hospital of Wuhan University. the head of National Natural Science Foundation of China, the Doctoral Fund of Ministry of Education of China and the Fundamental Research Funds for the Central Universities. The honor of French knight badge in 2006. Zheng LI as first author.
